# Biorealistic Control of Hand Prosthesis Augments Functional Performance of Individuals With Amputation

**DOI:** 10.3389/fnins.2021.783505

**Published:** 2021-12-14

**Authors:** Qi Luo, Chuanxin M. Niu, Chih-Hong Chou, Wenyuan Liang, Xiaoqian Deng, Manzhao Hao, Ning Lan

**Affiliations:** ^1^Laboratory of Neurorehabilitation Engineering, School of Biomedical Engineering, Shanghai Jiao Tong University, Shanghai, China; ^2^Institute of Medical Robotics, Shanghai Jiao Tong University, Shanghai, China; ^3^Department of Rehabilitation Medicine, Ruijin Hospital, School of Medicine, Shanghai Jiao Tong University, Shanghai, China; ^4^National Research Center for Rehabilitation Technical Aids, Beijing, China; ^5^Guangdong Work Injury Rehabilitation Hospital, Guangzhou, China

**Keywords:** biorealistic control, neuromuscular reflex, neuromorphic computation, tendon-driven prosthesis, hand grasp

## Abstract

The human hand has compliant properties arising from muscle biomechanics and neural reflexes, which are absent in conventional prosthetic hands. We recently proved the feasibility to restore neuromuscular reflex control (NRC) to prosthetic hands using real-time computing neuromorphic chips. Here we show that restored NRC augments the ability of individuals with forearm amputation to complete grasping tasks, including standard Box and Blocks Test (BBT), Golf Balls Test (GBT), and Potato Chips Test (PCT). The latter two were more challenging, but novel to prosthesis tests. Performance of a biorealistic controller (BC) with restored NRC was compared to that of a proportional linear feedback (PLF) controller. Eleven individuals with forearm amputation were divided into two groups: one with experience of myocontrol of a prosthetic hand and another without any. Controller performances were evaluated by success rate, failure (drop/break) rate in each grasping task. In controller property tests, biorealistic control achieved a better compliant property with a 23.2% wider range of stiffness adjustment than that of PLF control. In functional grasping tests, participants could control prosthetic hands more rapidly and steadily with neuromuscular reflex. For participants with myocontrol experience, biorealistic control yielded 20.4, 39.4, and 195.2% improvements in BBT, GBT, and PCT, respectively, compared to PLF control. Interestingly, greater improvements were achieved by participants without any myocontrol experience for BBT, GBT, and PCT at 27.4, 48.9, and 344.3%, respectively. The functional gain of biorealistic control over conventional control was more dramatic in more difficult grasp tasks of GBT and PCT, demonstrating the advantage of NRC. Results support the hypothesis that restoring neuromuscular reflex in hand prosthesis can improve neural motor compatibility to human sensorimotor system, hence enabling individuals with amputation to perform delicate grasps that are not tested with conventional prosthetic hands.

## Introduction

The loss of upper limbs hinders the ability of individuals with amputation to perform daily activities. Prosthetic hands are expected to restore lost hand functions based on the premise that the prosthesis would reproduce the similar motor consequences as a normal hand when accomplishing a task (Light et al., 2002). However, a high percentage of individuals with amputation tend to refuse myoelectric prosthetic hands due to difficulty in control (Atkins et al., 1996; Biddiss and Chau, 2007; McFarland et al., 2010). The functionality of modern prosthetic hands is still grossly inferior compared to the dexterity of human hand. Especially, a great challenge arises when controlling prosthetic devices to interact with real-world objects that are deformable or crispy. In such cases, the prosthetic hand is expected to adapt its compliance commensurate with object stiffness as the human hand does (Balasubramanian and Santos, 2014; Zhang et al., 2021). Therefore, we proposed that it is necessary to reanimate compliant property of human sensorimotor control in prosthetic hands (Lan et al., 2021).

The human hand achieves fine grasp control through the sensorimotor system with a series of physiological processes (Lemon, 2008). Motor intention is executed from recruitment of spinal motoneurons (Henneman et al., 1965), to generation of muscle forces to move joints (Bernstein, 1967; McNeill Alexander, 2002; Valero-Cuevas et al., 2007), and finally the formation of proprioceptive feedback to inform the brain of motor consequences (Weiler et al., 2019; Prochazka, 2021). An emergent approach to reanimating these physiological processes focuses on developing computational models that capture biologically realistic properties of sensorimotor system (Hodgkin and Huxley, 1952; Rack and Westbury, 1969; Zajac, 1989; Izhikevich, 2003; Mileusnic et al., 2006; Song et al., 2008; He et al., 2013; Li et al., 2015). A key step to achieve biorealistic control of prosthetic hands in real-time is made by implementing biorealistic models in neuromorphic chips capable of real-time computing (Niu et al., 2014, 2017). Biorealistic control may allow the prosthesis to match the behaviors of sensorimotor system of individuals with amputation (Niu et al., 2021; Zhang et al., 2021).

In our recent work, a model-based biorealistic controller (BC) inspired by neuromuscular reflex control (NRC) was developed using fast computing, neuromorphic technology (Niu et al., 2021). The BC contained physiologically realistic models of neuromuscular reflex, including a Hill-type muscle model (Hill, 1938), a spindle model (Mileusnic et al., 2006), an Izhikevich alpha-motoneuron pool (Izhikevich, 2003), and a monosynaptic reflex loop (Marsden et al., 1976; Hultborn, 2006). The force-control capability of the BC was evaluated, demonstrating potential applicability for prosthetic control (Luo et al., 2021). It was further revealed in a virtual hand that the finger stiffness with NRC can be automatically adjusted to fit that of grasped spring (Zhang et al., 2021). These studies established that the BC possesses human-like capabilities for force and stiffness control, which are fundamental qualities of human sensorimotor system (Salisbury and Craig, 1982; Johansson and Westling, 1988; Carter et al., 1990; Ettema and Huijing, 1994).

The goal of this study was to further explore to what extent the BC may enable individuals with amputation to better accomplish functional grasping tasks. Standard Box and Blocks Test (BBT), a Golf Balls Test (GBT), and a Potato Chips Test (PCT) were conducted to assess functional improvements. The latter two tasks were more challenging, and novel to prosthetic tasks. We hypothesized that restoring neuromuscular reflex could allow the prosthetic hand to behave like a human hand, therefore, enhancing the neural motor compatibility and facilitating individuals with amputation to control delicate grasps. Here the performance of BC with NRC was compared to a baseline proportional linear feedback (PLF) controller in individuals with amputation. The findings allowed us to understand how various factors, such as controller compliance, task difficulty, and experience of myocontrol, may influence functional performance of prosthetic hands.

## Materials and Methods

### Subjects

Eleven individuals with forearm amputation (ten males and one female, age range: 29–62 years) participated in the study. The detailed descriptions of participants are listed in Table 1. Subjects had no history of neurological disorders. This study was approved by the Ethics Committee of Human and Animal Experiments of the Med-X Research Institute of Shanghai Jiao Tong University. All participants gave written consent before joining the study.

**TABLE 1:** Clinical information of individuals with amputation.

Participant	Age	Sex	Amputation level and side	Year since amputation	Cause	Own prosthesis	Frequency of use	Dominant hand	Myoelectric prosthesis experience
S01	51	M	Left distal third of left forearm (long residual limb)	36	Trauma	Functionality	Daily	R	Yes
S02	40	M	Right distal third of right forearm (middle residual limb)	16	Trauma	Cosmetic	Daily	R	None
S03	46	M	Left distal third of left forearm (long residual limb)	13	Trauma	None	None	R	None
S04	29	M	Right distal third of right forearm (long residual limb)	2	Trauma	Functionality	Daily	R	Yes
S05	52	M	Right distal third of right forearm (long residual limb)	6	Trauma	Functionality	Daily	R	Yes
S06	45	M	Right distal third of right forearm (long residual limb)	22	Trauma	None	None	R	None
S07	55	M	Right distal third of right forearm (long residual limb)	8	Trauma	Functionality	Daily	R	Yes
S08	62	M	Right distal third of right forearm (long residual limb)	34	Trauma	Cosmetic	Daily	R	None
S09	38	F	Right distal third of right forearm (long residual limb)	5	Trauma	Functionality	Daily	R	Yes
S10	32	M	Right distal third of right forearm (long residual limb)	22	Trauma	Functionality	Daily	R	Yes
S11	32	M	Bilateral distal third of bilateral forearm (long residual limb)	23	Trauma	None	None	R	None

### Model-Based Biorealistic Controller

**FIGURE 1:**
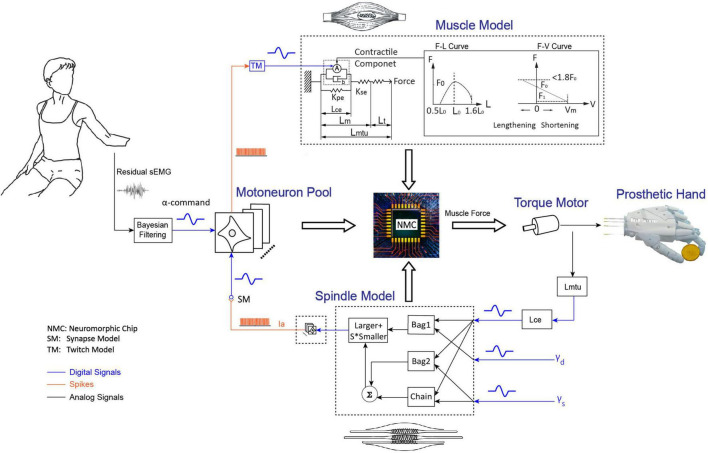
Integrated human and prosthetic hand system with biorealistic controller. Residual sEMG from the individual with amputation is decoded into alpha motor command to establish a model-calculated force for torque motor. Torque by the motor pulls a cable to create prosthetic hand movements. Proprioceptive information is deduced from rotation of torque motor as input to the biorealistic spindle. The neuromorphic model included a motoneuron pool with 768 spiking neurons, a skeletal muscle, and a muscle spindle projecting 128 spiking Ia afferents. The muscle length (*L_m*) and fascicle length (length of contractile element, *L*_*ce*_) are the key inputs to the muscle model and spindle model, respectively. The length of musculotendinous unit (*L*_*mtu*_) refers to the sum of *L_m* and the length of tendon (*L_t*). *L_m* refers to the sum of *L*_*ce*_ and the length of serial elastic element (*L*_*se*_). *F_0* is muscle active force when muscle contraction speed is zero, *F_1* is active force when muscle contraction speed is maximum (*V_m*). Spindle model consists of three intrafusal fibers (bag1, bag2, and nuclear chain), it receives 3 inputs (*L*_*ce*_, γ_*d*_, and γ_*s*_) to produce primary afferent firing (Ia). *S* is constant (*S* = 0.156).

The model-based BC was developed with programmable Very-Large-Scale-Circuit (VLSC) hardware (Figure 1; Niu et al., 2021). The neuromorphic chip integrated key models of NRC, which included a motoneuron pool, a skeletal muscle, and an associated muscle spindle. Electromyography (EMG) signal from residual muscle of the individual with amputation was filtered into alpha motor command by adopting a non-linear Bayesian algorithm (Sanger, 2007). Alpha motor command is the entrance to the BC. The motor command enters the biorealistic reflex loop as an excitatory postsynaptic current (EPSC), which is distributed to several pools of spiking motoneurons. Spiking outputs from the motoneurons are subsequently converted to excitatory drive to Hill-type model of skeletal muscle. The muscle model calculates a muscle force that will eventually be established by the torque motor. Information about muscle lengthening is sent back to a model of muscle spindle, which subsequently produces excitatory afferents that loop back to the motoneuron pool. For the BC, the reflex gain was set at 10% that the stiffness change was about 10% of baseline values with a small change of muscle fascicle length (Niu et al., 2021). This sufficed to produce realistic reflex behavior on a human cadaver hand (Niu et al., 2017). The reflex gain of controller can be modulated by changing the sensitivity of muscle spindle, which can be regulated by the gamma fusimotor inputs (gamma static and gamma dynamic).

The Hill-type muscle model converts alpha-motoneuron spikes into muscle force, depending on the muscle’s temporal length and lengthening velocity (Hill, 1938). Active force is caused by the contractile elements in a muscle through the actin and myosin ratcheting mechanism. The active force has been scaled with respect to the length of muscle in the muscle model. The Hill-type model uses standalone mechanical components approximate features during muscle contraction, e.g., force production, viscoelasticity, sudden release, etc. Spiking neurons are implemented following the Izhikevich model (Izhikevich, 2003), which takes the postsynaptic neural current as the input and produces a spike train as the output. In the BC, 768 alpha-motoneurons were divided into six pools with six various sizes. Since each motoneuron pool contained 128 neurons, their EPSCs were superimposed with independent random noises to allow 128 neurons to fire at similar rates but with different timing. The number of motoneurons was determined by balancing the innervation number of a typical mammalian muscle (Buchthal and Schmalbruch, 1980) and the maximum number allowed by the hardware (Niu et al., 2017).

Biorealistic proprioceptive feedback is provided by implementing the physiologically realistic model of muscle spindle (Mileusnic et al., 2006). The spindle model senses the information about muscle lengthening, and then produces excitatory afferents that loop back to the motoneuron pool. Based on this model, we emulated bag1, bag2, and nuclear chain fibers in a spindle, which produced Group Ia and Group II afferents according to its gamma fusimotor drives and muscle states. In order to ensure the asynchrony of spiking outputs from muscle spindle, independent random noises were added to the 128 spiking afferents. All spiking afferents were then fed back to the motoneurons with Group Ia afferents. However, only Group Ia afferents were included in the feedback loop, because the connections from Group II were not as clearly defined in monosynaptic circuitry.

### Baseline Controller for Comparison

**FIGURE 2:**
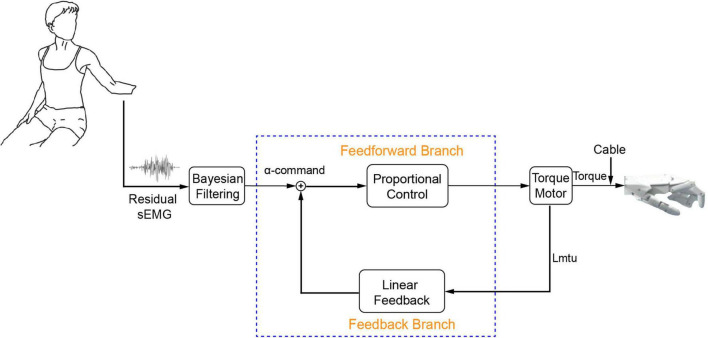
Proportional linear feedback (PLF) controller. It is used as a baseline controller for comparison purpose.

As shown in Figure 2, a PLF controller was used as a baseline controller for comparison with the BC. Closed-loop control was achieved by superimposing the alpha motor command and linear feedback. The feedback gain was 10%. The linear feedback converted length change of driven-cable to a feedback motor command, and the length change was measured by a rotational transducer on the torque motor. Gains of the feedforward branch and feedback branch were determined in our prior study (Luo et al., 2021). Proportional control was chosen here as baseline controller because it had been used in commercial prostheses (Farina et al., 2014; Segil and Weir, 2014). Linear feedback was added to model a simplified version of proprioceptive feedback in the baseline controller. This allowed assessing the comparative effect of feedback regulation with and without biorealistic proprioception.

### Cable-Driven Prosthetic Hand

The cable-driven prosthetic hand developed in prior work (Niu et al., 2021) was controlled by the BC or PLF controller to conduct grasp tasks. It is not a mature prosthetic hand or anywhere close to be a home-use medical device, but a research device that minimizes cost using an under-actuated design with a single torque motor (PD2-C42, Nanotec Electronic GmbH & Co. KG, Germany). The cables were attached to the shaft of motor. The motor generated a torque to cause cable tensioning, resulting in digits closing simultaneously according to a synergistic pattern. The raw surface EMG signals were sampled at rate of 1,962 Hz by Delsys system (Trigno Wireless EMG System, Delsys Inc., United States) from residual muscle contraction of individuals with amputation. The hand was 3D-printed and assembled by adopting an open-source design of cable-driven (InMoov) (InMoov, 2012). For all participants, a universal prosthetic socket was built, and the prosthetic socket could be adjusted by a forearm adapter according to the arm length of individuals with amputation. To reduce the burden of weight on participants wearing the prosthetic hand, the torque motor was moved proximally toward the elbow.

### Controller Property Test

Force control ability (Wen et al., 2020; Luo et al., 2021) and stiffness adjustment ability (Okuno et al., 2005; Zhang et al., 2021) are critical for grasp functions of the prosthetic hand. We designed the force variability test and stiffness property test to show the benefits of compliant control for controllers with and without biological properties.

#### Force Variability Test

**FIGURE 3:**
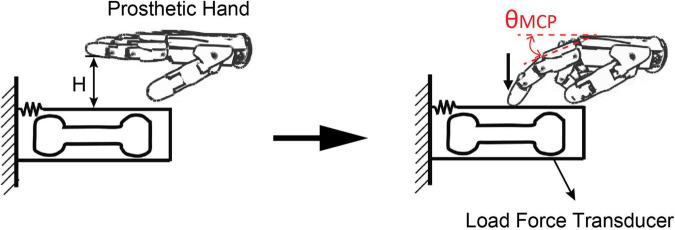
Experimental setup of force variability test for the two controllers. The prosthetic hand was fully extended at the beginning. Pseudo-random alpha commands were issued to the controller, which drove the prosthetic finger to move till the fingertip contact the force transducer.

The force variability of prosthetic hand with different controllers was tested using a finger pressing task. The index finger of the prosthetic hand was activated to press down a force transducer. As shown in Figure 3, the prosthetic finger was initially hovering at 1 cm above the force transducer (*H* = 1 cm). The prosthetic hand was fully extended at the beginning. Thereafter, pseudo-random alpha commands were issued to the prosthetic controller, which drove the prosthetic finger to move till the fingertip contact the force transducer. The fingertip force and metacarpophalangeal (MCP) joint angle of the prosthetic finger were recorded in real-time.

#### Stiffness Property Test

**FIGURE 4:**
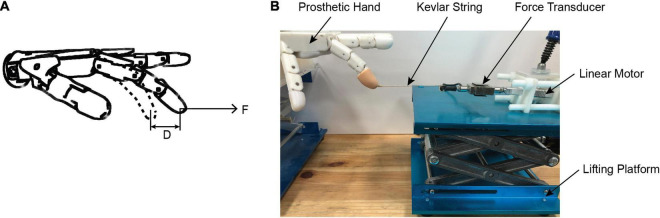
Stiffness test experiment. **(A)** Schematic diagram of stiffness test; **(B)** stiffness test platform for the prosthetic hand. A step alpha command was issued to the prosthetic controller, which drove the index finger to move to a specified position (chosen gesture). A linear motor to pull the finger in a chosen gesture to move 1 cm horizontally outward by the Kevlar string. The resistance (tensile force) of the finger to the string is measured by a force transducer fixed on the linear motor shaft. The stiffness of the finger can be calculated by the recorded force change and the distance of the finger movement.

As shown in Figure 4, we designed an experiment to test the stiffness adjustment range of the prosthetic hand with different controllers. Specifically, the prosthetic controller received an alpha motor command such that the index finger moved to a specified position (chosen gesture). We used a linear motor to pull the finger in a chosen gesture to move 1 cm horizontally. The resistance (tensile force) of the finger to the cable was measured by a tension sensor fixed on the linear motor shaft. Thus, the stiffness of the finger can be calculated by the recorded force change and the distance of the finger movement (*D* = 1 cm).

### Functional Task Test

To further assess and compare the performance and reliability of the BC in the functional tasks, participants were instructed to perform the following three functional tests: (1) BBT, (2) GBT, and (3) PCT. The three tasks represented increased difficulty and delicacy for a hand prosthesis to handle simulating daily living activities.

#### Box and Blocks Test

**FIGURE 5:**
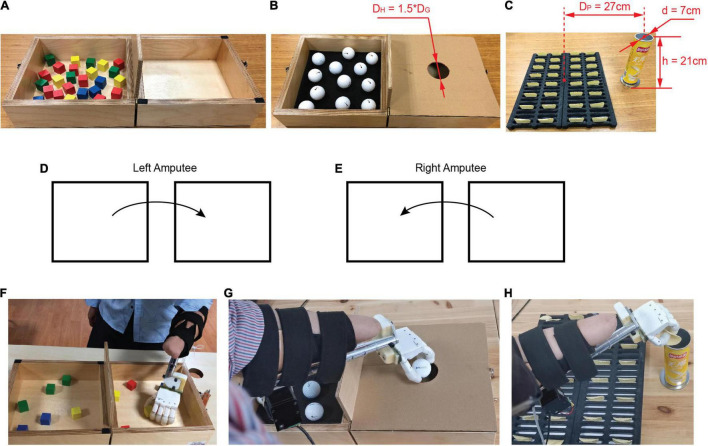
Overview of experimental setup. **(A)** Box and Blocks Test. **(B)** Design of Golf Balls Test. *D*_*G*_ represents the diameter of golf ball, and *D*_*H*_ denotes the diameter of hole in the middle of the lid. **(C)** Design of Potato Chips Test. *D*_*P*_ represents the distance between the center of slot and the center of storage tank. *d* and *h* denote, respectively the bottom diameter and height of storage tank. **(D)** For individuals with left amputation, they are instructed to transfer the objects from left to right. **(E)** For individuals with right amputation, they are instructed to transfer the objects from right to left. **(F)** Experimental scene in the Box and Blocks Test. **(G)** Experimental scene in the Golf Balls Test. **(H)** Experimental scene in the Potato Chips Test.

The first grasping task was the BBT (Mathiowetz et al., 1985), which required participants to transfer as many blocks as possible from one box to another in 1 min (Figures 5A,F). This test was standard for transfer of rigid objects. In the BBT, the number of square blocks that subjects were able to move from one box to another in 1 min was counted. If two blocks were picked up at a time, they would be counted as one. The number of successful transfers and that of drops were recorded as outcome measures.

#### Golf Balls Test

The GBT required more stable grasp and precise transfer of slippery objects, and was derived from the BBT. The test replicated the BBT, except that golf balls were used instead of the standard wooden ones and a lid with circular hole was set to cover the box on the side of the partition (Figures 5B,G). The hole is located in the middle of the lid, and its diameter (*D*_*H*_) is 1.5 times that of a golf ball (*D*_*G*_ = 42 mm). The weight of a golf ball is approximately 45 g. Participants were instructed to transfer as many golf balls as they could from one side of the partition to the other through the hole in 1 min, and this was counted as one run of the task. To prevent golf balls from rolling in the box, we placed a 1 cm thick sponge at the bottom of the box. The performance was measured by the number of successful transfers and that of drops during the 1 min test, similar to the BBT.

#### Potato Chips Test

We designed a novel test that is the task of picking potato chips in daily life. The PCT demanded delicate grasp and precise transfer of brittle objects. As shown in Figure 5C, the potato chips are placed vertically in a customized slot, and the storage tank of potato chips is fixed on the side of the placement slot. The distance between the center of the slot and the center of the storage tank (*D*_*P*_ = 27 cm) is equal to the distance between the centers of the boxes on both sides of the partition in the BBT. The diameter of the storage tank is ∼7 cm and the height is ∼21 cm. Participants were instructed to transfer the potato chips from the placement slot to the storage tank as fast as possible but without breaking them in 1 min (Figure 5H). The performance was measured by the number of successful transfers, number of drops, and that of breaks in the 1 min test. In order to maintain consistency, the canned original Lay’s potato chips were uniformly used in the experiment.

### Experimental Protocol

To evaluate the performance of prosthetic controllers, an experimental protocol was designed following the two parts. First, we conducted the controller property tests on the prosthetic hand without human operation.

Second, subjects were instructed to manipulate a prosthetic hand with two controllers (BC, PLF) to complete a series of functional tasks that were introduced in the order of increasing complexity: first the BBT task, then the GBT task, and finally the PCT task. For individuals with left amputation, they were instructed to transfer the objects from left to right (Figure 5D), and for individuals with right amputation, they were instructed to transfer the objects from right to left (Figure 5E). Subjects were familiarized with the tasks and accustomed to the controllers for about 20 min. During training time, participants were competent to manipulate the prosthetic hand for grasping tasks. To avoid fatigue, participants were allowed 1 min of rest between tests. Furthermore, there was a 10-min break between the three tasks. Participants performed each task with five repetitions for each controller. For each task, a block design was adopted based on controllers, i.e., block 1 for BC and block 2 for PLF. The sequence of blocks and the tests within a block were randomized. The whole functional test lasted approximately 2 h.

### Index of Difficulty of Task

The Index of Difficulty of Task (*ID*_*Task*_) was defined as the square root of the sum of the number of drops and the number of breaks divided by the number of successful transfers. The calculation equation is as follows:


(1) $$I\,D_{T\,a\,s\,k}=\sqrt{\frac{N_{D\,r\,o\,p}+N_{B\,r\,e\,a\,k}}{N_{S\,u\,c\,c\,e\,s\,s}}}$$

where *N*_*Success*_ is the average number of successful transfers for all subjects, *N*_*Drop*_ is the average number of drops for all subjects, and *N*_*Break*_ is the average number of breaks for all subjects in each task.

### Outcome Measures

#### Measures in Controller Property Tests

1. Variability of force
We assumed that minimizing force variability was equated to minimizing the root-mean-square (RMS) of *F*′ (force derivative, *FD*), which was the time derivative of the applied force (*F*). The RMS of *FD* was calculated as follows (Flash and Hogan, 1985):

(2) $$F\,D_{R\,M\,S}=\sqrt{\frac{1}{T}\,\sum_{t=1}^{t=T}{\left(F^{\prime }\,\left(t\right)\right)}^{2}}$$

where *FD*_*RMS*_ denoted the variability of applied force, and *T* stands for the force’s duration.
2. Variability of joint angle
We assumed that minimizing joint angle variability was equated to minimizing the RMS of *JA*″ (second derivative of joint angle, *JAD*), which was the time second derivative of the joint angle (*JA*). The RMS of *FD* was calculated as follows (Flash and Hogan, 1985):

(3) $$J\,A\,D_{R\,M\,S}=\sqrt{\frac{1}{T}\,\sum_{t=1}^{t=T}{\left(J\,A^{{}^{\prime \prime }}\,\left(t\right)\right)}^{2}}$$

where *JAD*_*RMS*_ denoted the variability of joint angle of the prosthetic finger, and *T* stands for the duration.
3. Stiffness adjustment range
The stiffness of the prosthetic finger (*K*_*stiffness*_) can be calculated by the force change (*F*_*C*_) and the distance of the finger movement (D = 1cm). The calculation was expressed as follows:

(4) $$K_{s\,t\,i\,f\,f\,n\,e\,s\,s}=\frac{F_{C}}{D}$$

The stiffness adjustment range (*KAD*_*stiffness*_) was equal to the maximum stiffness (*K*_*max*_) minus the minimum stiffness (*K*_*min*_), as follows:

(5) $$K\,A\,D_{s\,t\,i\,f\,f\,n\,e\,s\,s}=K_{m\,a\,x}-K_{m\,i\,n}$$

#### Measures in Functional Tests

1. Number of successful transfers (*N*_*Success*_)
The number of successfully transferred objects (blocks/golf-balls/potato-chips) during the 1 min test was used as outcome measure.
2. Number of drops (*N*_*Drop*_)
The number of dropped objects (blocks/golf-balls/potato-chips) during the 1 min test was used as outcome measure.
3. Number of breaks (*N*_*Break*_)
The number of broken objects (potato-chips) during the 1 min test was used as outcome measure.
4. Comprehensive metric of performance (CMP)
The comprehensive metric performance of participants was defined as the number of successful transfers (*N*_*Success*_) minus the number of drops (*N*_*Drop*_) and the number of breaks (*N*_*Break*_) during the 1 min test. It is calculated as follows:

(6) $$C\,M\,P=N_{S\,u\,c\,c\,e\,s\,s}-N_{D\,r\,o\,p}-N_{B\,r\,e\,a\,k}$$

5. Difference in controller performance (DCP)
The percentage of difference in performance of BC and PLF controllers was defined as follows:

(7) $$D\,C\,P=\frac{C\,M\,P_{B\,C}-C\,M\,P_{P\,L\,F}}{\left|C\,M\,P_{P\,L\,F}\right|}\times 100\%$$

### Statistical Analysis

One-way repeated measures analysis of variance (ANOVA) was performed to estimate the effect between the controllers for all participants. And two-way repeated measures ANOVA was used to detect the effects of controller and myocontrol experience on outcome measures. *Post hoc* comparisons with a Bonferroni-corrected assessed the pairwise differences between controllers or myocontrol experience. Data processing was done using MATLAB (R2014b, MathWorks Inc., Natick, MA, United States). All averages are reported by mean ± standard deviation (Mean ± SD). All statistical analysis was carried out with R, version 4.1.0.

## Results

### Compliant Properties of Controllers

**FIGURE 6:**
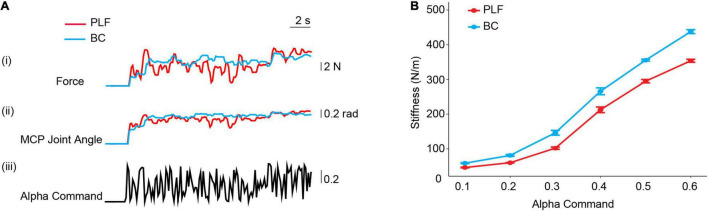
**(A)** Force and position responses of the prosthetic hand with BC and PLF controllers pressing a force transducer. (i) Fingertip force of prosthetic hand; (ii) MCP joint angle of prosthetic hand; (iii) pseudo-random inputs of alpha motor commands. **(B)** Stiffness property of controllers. Varying alpha motor commands produced different stiffness in two controllers (PLF, BC). The stiffness adjustment range of the prosthetic hand with BC and PLF is determined with a perturbation force applied to fingertip.

Force and position responses of the prosthetic hand pressing a force transducer are shown in Figure 6A. When the controllers received alpha commands, the prosthetic finger started to move, first the MCP joint angle changed, and then the prosthetic finger contacted the object to generate a force. As alpha motor commands were changing in real-time, the MCP joint angle and fingertip force of the prosthetic hand also made changes. Compared with PLF control, the fingertip force and MCP joint angle changes of the prosthetic hand using BC were more gradual. The force variability (*FD*_*RMS*_) of using BC was 49.8% lower than that of PLF control, and the variability of MCP joint angle (*JAD*_*RMS*_) was reduced by 19.6%.

Figure 6B shows that the prosthetic finger can generate different stiffness with different alpha motor commands. With averaged results across 10 measurements, stiffness of the prosthetic hand with BC varied with a wider range (55.6–447.9 N/m) than that of PLF (44.7–363.0 N/m). Compared to PLF, the stiffness adjustment range (*KAD*_*stiffness*_) with BC was improved by 23.2%.

### Performance in Box and Blocks Test

**FIGURE 7:**
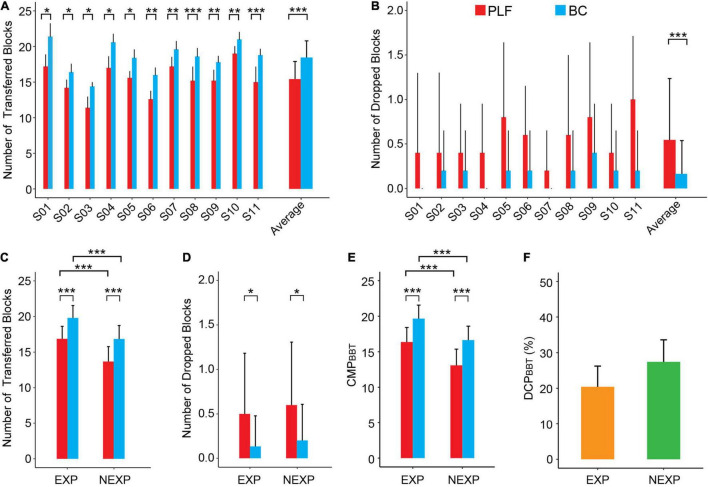
Performance metrics of participants in the BBT. **(A)** The number of successfully transferred blocks for each participant. **(B)** The number of dropped blocks for each participant. **(C,D)** Average results for the outcome measures across different experimental conditions (BC/PLF; EXP/NEXP). **(E,F)** Average results for the CMP and DCP in EXP and NEXP groups (*p < 0.05; **p < 0.01; ***p < 0.001).

Performance of 5 sessions of the BBT task by 11 participants is shown in Figure 7. For each subject, the number of successfully transferred blocks with BC was significantly more than that with PLF (Figure 7A). But there was no significant difference in the number of dropped blocks (Figure 7B). In all subjects, the BC significantly outperformed PLF (Figures 7A,B) with an average number of successful transfers of 18.45 ± 2.34 by BC vs. 15.42 ± 2.48 by PLF (*p* < 0.001) and a very small number of dropped blocks by BC (0.16 ± 0.37) vs. PLF (0.55 ± 0.69; *p* < 0.001).

When comparing the performance of subjects with myocontrol experience (EXP) to those of non-experience (NEXP) in myocontrol, the number of transferred blocks in EXP group was significantly more than that in NEXP group (*p* < 0.001; Figure 7C), and so was the comprehensive metric of performance (CMP_*BBT*_; *p* < 0.001) as shown in Figure 7E. But there was no statistically significant difference in the number of dropped blocks (*p* = 0.459; Figure 7D). In both groups, BC consistently outperformed PLF, and experience with myocontrol did have a significant effect on controller performance in BBT task (Figure 7E). However, the difference in controller performance (DCP_*BBT*_) was not significant between EXP and NEXP groups (*p* = 0.085; Figure 7F).

### Performance in Golf Balls Test

**FIGURE 8:**
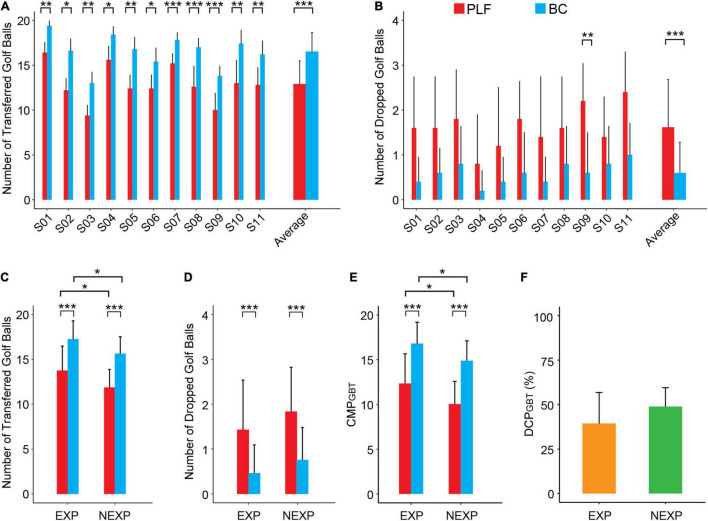
Performance metrics of participants in the GBT. **(A)** The number of successfully transferred golf balls for each participant. **(B)** The number of dropped golf balls for each participant. **(C,D)** Average results for the outcome measures across different experimental conditions (BC/PLF; EXP/NEXP). **(E,F)** Average results for the CMP and DCP in EXP and NEXP groups (*p < 0.05; **p < 0.01; ***p < 0.001).

Results of 5 sessions of the GBT task for all participants are reported in Figure 8. For each subject, the number of successfully transferred golf balls with BC was significantly more than that with PLF (Figure 8A). In addition, except for S09, there was no significant difference in the number of dropped blocks (Figure 8B). In all subjects, the BC significantly outperformed PLF (Figures 8A,B) with an average number of successful transfers of 16.53 ± 2.12 by BC vs. 12.91 ± 2.58 by PLF (*p* < 0.001), and a small number of dropped golf balls by BC (0.60 ± 0.68) vs. PLF (1.62 ± 1.06; *p* < 0.001).

For performance of subjects with EXP to those with NEXP, the number of transferred golf balls in EXP group was significantly more than that in NEXP group (*p* < 0.05; Figure 8C), and so was the CMP_*GBT*_ (*p* < 0.05; Figure 8E). But no significant difference in the number of dropped blocks was apparent (*p* = 0.051; Figure 8D). In both groups, BC consistently outperformed PLF, and experience with myocontrol also affected controller performance significantly in GBT task (Figure 8E). Nevertheless, the difference in controller performance (DCP_*GBT*_) was not significant between EXP and NEXP group (*p* = 0.288; Figure 8F).

### Performance in Potato Chips Test

**FIGURE 9:**
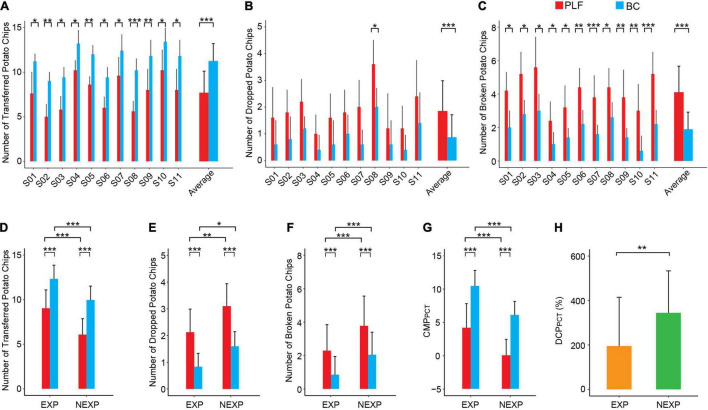
Performance metrics of participants in the PCT. **(A)** The number of successfully transferred potato chips for each participant. **(B)** The number of dropped potato chips for each participant. **(C)** The number of broken potato chips for each participant. **(D–F)** Average results for the outcome measures across different experimental conditions (BC/PLF; EXP/NEXP). **(G,H)** Average results for the CMP and DCP in EXP and NEXP groups (*p < 0.05; **p < 0.01; ***p < 0.001).

In 5 sessions of the PCT task, controller performance is more variable as shown in Figure 9. For each subject, the number of successfully transferred potato chips with BC was significantly more than that with PLF (Figure 9A). Also, there was no significant difference in the number of dropped potato chips except for S08 (Figure 9B). But the number of broken potato chips with BC was significantly less than PLF (Figure 9C). In all subjects, the BC significantly outperformed PLF again (Figures 9A–C) with an average number of successful transfers of 11.25 ± 1.95 by BC vs. 7.69 ± 2.43 by PLF (*p* < 0.001), a small number of dropped potato chips by BC (0.87 ± 0.84) vs. by PLF (1.85 ± 1.13; *p* < 0.001), and a number of broken potato chips by BC (1.89 ± 1.03) vs. by PLF (4.11 ± 1.56; *p* < 0.001).

When examining the performance of subjects with EXP to those with NEXP, the number of transferred potato chips in EXP group was significantly more than that in NEXP group (*p* < 0.001; Figure 9D), and so was CMP_*PCT*_ (*p* < 0.001; Figure 9G). Furthermore, the number of dropped potato chips in EXP group was significantly less than that in NEXP group (*p* < 0.05; Figure 9E), and so was the number of broken potato chips (*p* < 0.001; Figure 9F). In both groups, BC consistently outperformed PLF, and the effect of experience with myocontrol on controller performance was significant (Figure 9G). But interestingly, the difference in controller performance (DCP_*PCT*_) in EXP group was significantly lower than that in NEXP group (*p* < 0.01) as shown in Figure 9H. This suggested that novel subjects without any myocontrol experience (NEXP group) may adapt biorealistic control more rapidly to yield a greater improvement in performance than the EXP group in the PCT task.

### Overall Analysis of Performance

**FIGURE 10:**
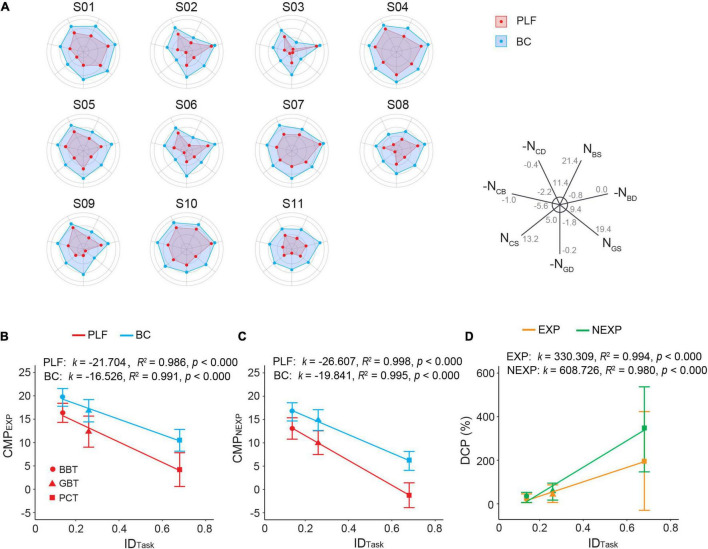
**(A)** Performance metrics overview of 11 participants with different controllers (BC, PLF) in the three functional tasks. *N*_*BS*_, number of blocks successfully transferred; *N*_*BD*_, number of blocks dropped; *N*_*GS*_, number of golf balls successfully transferred; *N*_*GD*_, number of golf balls dropped; *N*_*CS*_, number of potato chips successfully transferred; *N*_*CD*_, number of potato chips dropped; *N*_*CB*_, number of potato chips broken. For visualization purpose, measures of failed performance were plotted with negative scale, such that a larger value represented better performance. Minimal and maximal values were indicated in each axis. **(B)** Relationship between CMP and index difficulty of task (ID_Task_) in EXP group. **(C)** Relationship between CMP and index difficulty of task in NEXP group. **(D)** Relationship between DCP and ID_Task_ in EXP and NEXP groups. *k* represents slope of linear regression between Outcome Measure and ID_Task_.

In summary, performance of the BC exceeded that of the baseline PLF controller in three tasks of varying difficulties. Figure 10A shows the overall comparison of the controller performance in three functional tasks for all subjects in a spiderweb plot. the larger the web area, the better the performance. It clearly shows that in all 11 subjects, BC consistently outperformed PLF. Among the subjects, S01, S04, S05, S07, and S10 had larger performance areas with each controller. S03 seemed to give the worst amongst them. In general, increase in task difficulty led to a deterioration in performance measure (CMP) in both EXP and NEXP groups of subjects operating the two controllers (*p* < 0.001; Figures 10B,C). However, the decline in CMP with BC was slightly less compared with PLF (Figures 10B,C). To differentiate performance between BC and PLF, a performance disparity was defined in Eq. 7 to assess increase in CMP. It is interesting that the performance escalation characterized by DCP was larger in NEXP group compared with that in EXP group (Figure 10D), which was particularly significant for PCT task (*p* < 0.001; Figure 9H).

## Discussion

The purpose of this study is to understand how well the BC with restored neuromuscular reflex could facilitate individuals with amputation to control a cable-driven prosthetic hand. The underlying assumption highlights the roles of restored neuromuscular reflex in improving neural motor compatibility and grasp performance. Neural motor compatibility implies two aspects in human-prosthesis interaction. First, the prosthesis should have seamless continuity in motor control signals carrying motor intention from the human sensorimotor system; second, the prosthesis should produce a congruent motor action as the human sensorimotor system does (Lan et al., 2019). The issue of grasp control has been extensively addressed in prosthesis community (Okuno et al., 2005; Kuiken, 2009; Clemente et al., 2016; Hahne et al., 2018; Zhuang et al., 2019; Ortiz-Catalan et al., 2020; Piazza et al., 2020; Wen et al., 2020; Gu et al., 2021). However, the concept and importance of neural motor compatibility are only elaborated recently for prosthesis control (Lan et al., 2021). Intuitively, neural motor compatibility means that the more similar the prosthetic hand behaves as the human hand, the more effective individuals with amputation can control the prosthetic hand. In a pseudo-physiologic study, a virtual hand with restored NRC exhibits human-like compliant behaviors with a unique nature of stiffness adaption when grasping a soft object (Zhang et al., 2021). Results here further corroborates the teleological correlation between NRC and neural motor compatibility in hand prosthesis.

Results show that restored neuromuscular reflex in prosthesis transcends the performance of individuals with amputation in the three tasks. Participants using BC achieved an average improvement of 23.6, 43.7, and 263.8% of the CMP compared to the PLF controller in the BBT task, GBT task, and PCT task, respectively (Figure 10D). The remarkably improved performance in BC is attributed to restored neuromuscular reflex. The neuromorphic model repairs the disrupted neuro-mechanical process in the prosthetic hand, so that the prosthesis recaptures the unique non-linear biomechanics of human muscle with realistic force-length and force-velocity properties (Luo et al., 2021; Niu et al., 2021). This gives rise to the desirable compliance with stiffness and viscosity in the prosthetic hand (Rack and Westbury, 1969; Hoffer and Andreassen, 1981; Zhang et al., 2021). When the prosthetic hand adopts this property to grasp objects, the force exerted on the target object is more stable and less prone to random changes (Figure 6A). Muscle viscoelasticity is further regulated by local reflex circuits at the spinal cord to yield a wider range of stiffness adaptability (Crago et al., 1976; Hultborn, 2006). A compliant limb is, therefore, able to cope with unexpected disturbances during movement or force exertion, so that human sensorimotor system does not need to distinguish the two control states of movement or force during grasps (Hogan, 1984; Haken et al., 1985; Lan et al., 2019, 2021; Yang et al., 2019; Zhang et al., 2021). The similar behaviors to human sensorimotor control ease the cognitive burden on individuals with amputation to compensate the alien behaviors of prosthesis, thus, ameliorating grasp performance, more notably in delicate tasks such as PCT. These results provide strong supporting evidence for the hypothesis that restoring neuromuscular reflex could strengthen neural motor compatibility between prosthesis and sensorimotor system, which in turn augments the ability for prosthesis control of individuals with amputation.

It is noteworthy that from results of controller property tests, the force variability of biorealistic control was reduced by 49.8%, and the biorealistic control achieved a better compliant property with a 23.2% wider range of stiffness adjustment than that of the PLF control. A smoother (less variable) force and joint trajectory in response to a random “alpha command” indicate a steadier motor action that is less susceptible to disturbance. This is particularly important for delicate grasping of brittle objects. The ideal goal of stiffness control is to match the stiffness of object grasped (Lee et al., 1994; Zhang et al., 2021). A remarkable trait of neuromuscular reflex system is the ability to regulate its stiffness to adapt to external loading conditions. Therefore, a larger range of stiffness adjustment indicates an enhanced ability of compliant control, that is, it can grasp objects with a larger range of stiffness or softness. Thus, the range of stiffness adjustment implicates the capacity for functional performance. Functional tests revealed that the BC outperformed the baseline controller consistently. This is directly related to its smoother force control and better ability of stiffness regulation. Both properties contribute to a steadier grasp with less slippage and a more precise force exserted on objects, particularly for grasping more brittle potato chips. Compared with the PLF control, the biorealistic control yielded 263.8% improvement in CMP in the PCT task, which was much higher than the performance improvement in controller property tests (Figure 6). This suggests that BC with restored neuromuscular reflex made the prosthetic hand more compatible with individuals with amputation, which may result in a further improvement in grasping tasks.

Results indicate that task difficulty has a strong effect on controller performance. The BBT task is a standard experimental paradigm, but the GBT and the PCT are not found in literature (see a survey in Appendix). The three functional tasks are sorted in the order of increasing difficulty. Golf balls and potato chips are easy to slip or break (see Supplementary Movie 1). As shown in Figures 10B,C, the BBT task had the highest CMP score, followed by the GBT task, and the PCT task was the most challenging with the lowest CMP score. It is noted that with PLF control, there was a high rate of failure (slip or break) (Figures 9E,F), such that the average CMP score for PCT was below zero (Figure 10C). But encouragingly, the improvement in BC performance (DCP) was gradually larger as task difficulty increased (Figures 9H, 10D). This revealed that the advantage of BC could be more prominent in more challenging tasks. This offers convincing evidence that restoring neuromuscular reflex in hand prosthesis could approach the functionality of human hand.

Prior experience in using myocontrol of prosthesis may play a more subtle role in the performance of individuals with amputation. Experienced subjects generally performed better than those novel subjects with each controller in three tasks (Figure 7E, *p* < 0.001; Figure 8E, *p* < 0.05; Figure 9G, *p* < 0.001). But when examining the enhancement in controller performance in each subject, data revealed a significant difference between the EXP and NEXP groups (Figure 9H, *p* < 0.01) for the most difficult task (PCT), but no significant difference for the other two less difficulty tasks (BBT and GBT). It is counterintuitive that subjects in the NEXP group showed a greater tendence to improve performance with BC than those in the EXP group. In other words, novel subjects without any myocontrol experience could adapt BC control more rapidly than experienced subjects. In PCT task, the tolerance zone for grasp force is small. Since BC could maintain a smaller force variability than PFL (Figure 6A), it can, therefore, more successfully grasp and transfer frail potato chips. We speculate that subjects without myocontrol experience are more direct to learn to control the device; while experience with myocontrol could hinder subjects to adapt the BC that requires learning a new operation different from their prior experience. With the same amount of time given to all subjects to familiarize the new prosthesis at the beginning of experiment, prior experience may give these subjects a disadvantage in learning to control the new device. Even so, we expect that adequate training before using BC can allow all subjects to operate BC proficiently.

It was worth noting that before the tests, all subjects expressed doubt to complete the PCT task. However, after a short time of familiarization, they were quick to master the new prosthetic hand. Participants also could handle other delicate objects such as an egg and a grape (see Supplementary Movie 1). In other studies, delicate grasping was usually carried out in prosthetic hands integrated with tactile feedback (Tan et al., 2014; Micera et al., 2020; Ortiz-Catalan et al., 2020; Gu et al., 2021). Because of good neural motor compatibility, the BC demanded less cognitive effort from the user, and its motor action could occur with a minimal delay due to the ultra-high-speed real-time computation of neuromorphic hardware. Results presented here are encouraging on the efficacy, superiority, and adaptability of the BC for persons with forearm loss. A literature survey indicates that the performance of BC was better than that of most prosthetic devices in standard BBT task (see Table A3). Although torque control may be more prone to noise in the sEMG signals, both BC and PLF controllers displayed an adequate control for a torque motor in this study. Therefore, their abilities to smooth out noise in sEMG signals are manifested in their relative performance in the three tasks. How torque/force control may compare with a velocity control in conventional hand prosthesis remains to be elucidated in future studies.

This study focuses on integrating biorealistic models for restoring neuromuscular reflex and verifying the feasibility and effectiveness of the technology in practical application to prostheses. Several studies have established different muscle models (Hill, 1938; Rack and Westbury, 1969; Woittiez et al., 1984; Zajac, 1989; Biewener et al., 2014), neuron models (Hodgkin and Huxley, 1952; Rosenblatt, 1958; Izhikevich, 2003; Binczak et al., 2006; Hayati et al., 2016), and spindle models (Crowe, 1968; Rudjord, 1970; Schaafsma et al., 1991; Lin and Crago, 2002; Mileusnic et al., 2006). Among the muscle models, the Hill-type model is a widely accepted muscle model due primarily to its simplicity in computation. As for neuronal model, the Izhikevich model presents very natural patterns of firing (spiking) without much loss of biological realism compared to the acclaimed Hodgkin–Huxley model. To the best of our knowledge, the spindle model adopted in this study is the only available model that is the closest to its biological counterpart. A limitation in this study is that only one muscle was enabled in the cable-driven hand for flexion, leaving the extension passively stretched by a spring. This setup lacked the biological realism that both flexion and extension should be separately actuated by a pair of antagonistic muscles. The current prototype prevented the use of muscle co-contraction to increase system stiffness as humans do. Adding an antagonistic muscle in the BC would enable a direct modulation of system stiffness by modulating co-contraction of antagonists (Ajoudani et al., 2014). In addition, reflex gain of the controller could also be modified to modulate controller stiffness by adjusting fusimotor inputs to the spindle model *via* gamma static or dynamic activities. Although the assessment tasks conducted here showed promising benefits of proprioceptive afferent in functional grasping tasks for prosthetic hands, individuals with amputation mainly relied on visual feedback (Winges et al., 2003; Saunders and Knill, 2004) to guide the motion and to exert grasping force of the prosthetic hand. In the follow-up work, it is necessary to introduce tactile feedback into prosthetic hands (Tan et al., 2014; George et al., 2019; Zollo et al., 2019; Bensmaia et al., 2020; Hao et al., 2020; Micera et al., 2020; Farina et al., 2021; Raspopovic et al., 2021) to construct a fully biorealistic hand prosthesis (Lan et al., 2021). Further tests in a larger scale study with more users are required to confirm the long-term reliability, robustness, embodiment and acceptability in activities of daily living.

## Data Availability Statement

The raw data supporting the conclusions of this article will be made available by the authors, without undue reservation.

## Ethics Statement

The studies involving human participants were reviewed and approved by the Ethics Committee of Human and Animal Experiments of the Med-X Research Institute of Shanghai Jiao Tong University. The patients/participants provided their written informed consent to participate in this study.

## Author Contributions

QL, CN, and NL designed the experiment study and analyzed the experimental data. QL performed the experiment, prepared the figures and tables, and drafted the manuscript. C-HC provided the support for setting experimental platform. WL, XD, and MH recruited the individuals with amputation for this study. NL proposed the analytical method, and revised the manuscript. All authors contributed to the article and approved the submitted version.

## Conflict of Interest

The authors declare that the research was conducted in the absence of any commercial or financial relationships that could be construed as a potential conflict of interest.

## Publisher’s Note

All claims expressed in this article are solely those of the authors and do not necessarily represent those of their affiliated organizations, or those of the publisher, the editors and the reviewers. Any product that may be evaluated in this article, or claim that may be made by its manufacturer, is not guaranteed or endorsed by the publisher.

## Appendix

**TABLE A1:** Summary of outcome measures of functional test for participants.

Control scheme	Box and Blocks Test (BBT)		Golf Balls Test (GBT)		Potato Chips Test (PCT)		
	Success	Drop	Success	Drop	Success	Drop	Break
PLF	15.42 ± 2.48	0.55 ± 0.69	12.91 ± 2.58	1.62 ± 1.06	7.69 ± 2.43	1.85 ± 1.13	4.11 ± 1.56
BC	18.45 ± 2.34*	0.16 ± 0.37*	16.53 ± 2.12*	0.60 ± 0.68*	11.25 ± 1.95*	0.87 ± 0.84*	1.89 ± 1.03*

**Indicates a significantly better result than PLF control (p < 0.05).*

**TABLE A2:** Summary of outcome measures of functional test for participants with and without myocontrol experience.

Myocontrol experience	Control scheme	Box and Blocks Test (BBT)		Golf Balls Test (GBT)		Potato Chips Test (PCT)		
		Success	Drop	Success	Drop	Success	Drop	Break
With	PLF	16.87 ± 1.76	0.50 ± 0.68	13.77 ± 2.71	1.43 ± 1.10	9.03 ± 2.06	1.43 ± 0.97	3.40 ± 1.38
	BC	19.80 ± 1.75*	0.13 ± 0.35*	17.27 ± 2.03*	0.47 ± 0.63*	12.33 ± 1.54*	0.53 ± 0.68*	1.33 ± 0.80*
Without	PLF	13.68 ± 2.10	0.60 ± 0.71	11.88 ± 2.01	1.84 ± 0.99	6.08 ± 1.78	2.36 ± 1.11	4.96 ± 1.34
	BC	16.84 ± 1.91*	0.20 ± 0.41*	15.64 ± 1.89*	0.76 ± 0.72*	9.96 ± 1.57*	1.28 ± 0.84*	2.56 ± 0.87*

**Indicates a significantly better result than PLF control (p < 0.05).*

### Literature Survey

To analyze the performance of BC and PLF with a wider perspective, results are also compared with other studies in literature that use the standard BBT to evaluate performance of prosthetic hands and control strategies. Table A3 presents the average results for performance of participants and those of other prosthetic hands in literature. Note the literature survey may not be comprehensive. It includes only those prostheses of myoelectrical control.

**TABLE A3A:** Average results for individuals with amputation using BC and PLF.

Terminal device	Control scheme	Number of individuals with amputation Tested	BBT	GBT	PCT
Our cable-driven prosthetic hand	Biorealistic control	11	18.45 ± 2.34	16.53 ± 2.12	11.25 ± 1.95
	PLF control	11	15.42 ± 2.48	12.91 ± 2.58	7.69 ± 2.43

**TABLE A3B:** Performance of other prosthetic hands in literature.

Terminal device	Control scheme	Number of individuals with amputation Tested	BBT	GBT	PCT
Average performance		20	9.33 ± 4.90	NA	NA
SH2-P (Piazza et al., 2020)	Continuous pattern recognition control	3	10.67 ± 2.3	NA	NA
SH-P (Godfrey et al., 2018)	Direct control	9	9.6 ± 1.4	NA	NA
Motion control electric terminal device (Hebert and Lewicke, 2012)	Direct control	1	20	NA	NA
Hybrid device hand (Dromerick et al., 2008)	Switch-controlled (humeral abduction)	1	6	NA	NA
Ottobock 8E44 DMC plus (Dromerick et al., 2008)	Direct control	1	8	NA	NA
Michelangelo hand (Kuiken et al., 2016)	Direct control	3	14.7 ± 9	NA	NA
	Pattern recognition control	3	5.7 ± 1.2	NA	NA
Otto bock sensor-hand speed (Lenzi et al., 2016)	Direct control	1	3.8	NA	NA
RIC arm (Lenzi et al., 2016)	Pattern recognition control	1	5.5	NA	NA

## Abbreviations

NRC: neuromuscular reflex control
BBT: Box and Blocks Test
GBT: Golf Balls Test
PCT: Potato Chips Test
BC: biorealistic controller
PLF: proportional linear feedback
VLSC: Very-Large-Scale-Circuit
EMG: electromyography
MCP: metacarpophalangeal
*JAD_RMS_*: root-mean-square of second derivative of joint angle
*KAD_stiffness_*: stiffness adjustment range
EXP: experience of myocontrol
NEXP: non-experience of myocontrol
CMP: comprehensive metric of performance
DCP: difference in controller performance
*ID*_*task*_: index of difficulty of task
MVC: maximum voluntary contraction
*N*_*Success*_: number of successful transfers
*N*_*Drop*_: number of drops
*N*_*Break*_: number of breaks
*FD_RMS_*: root-mean-square of force derivative
ANOVA: analysis of variance.
